# High-concentrate diet decreases lamb fatty acid contents by regulating bile acid composition

**DOI:** 10.1016/j.fochx.2024.101871

**Published:** 2024-10-05

**Authors:** Kaizhi Zheng, Liangyong Guo, Yang Cao, Yuyang Yin, Hui Gao, Xiaowei Zhang, Junfang Jiang, Jinbing Li, Xin Huang, Kui Li, Sangang He

**Affiliations:** a Institute of Animal Husbandry and Veterinary, Zhejiang, Academy of Agricultural Sciences, Hangzhou 310021, China; b Animal Husbandry Technology Promotion and Breeding Livestock and Poultry Monitoring Station of Zhejiang Province, Hangzhou 310000, China; c Huzhou Academy of Agricultural Sciences, Huzhou 313000, China; d Shangyu District Animal Husbandry and Veterinary Technology Promotion Center, Shaoxing 312300, China

**Keywords:** High-concentrate diet, Metabolomics, Lamb, Bile acid, Fatty acid

## Abstract

Feeding sheep with high-concentrate diet (HCD) to shorten production cycle is a well-developed feeding strategy to increase lamb production. Here, metabolomics were performed to explore the mechanism that HCD changes lamb nutrition composition. Differential metabolites were enriched in primary bile acid biosynthesis. Significantly higher content of bile acids including taurodeoxycholic acid sodium salt (TDCA), taurochenodeoxycholic acid sodium salt (TCDCA) and taurocholic acid (TCA) was observed in lamb of HCD, while the content of lithocholic acid (LCA), cholic acid (CA), chenodeoxycholic acid (CDCA) and Chenodeoxycholic acid-3-beta-D-glucuronide (CDCA-3Gln) were higher in the controls. Furthermore, a significantly decreased content of fatty acids was observed in lamb of HCD group. Finally, primary skeletal cells treated with CA or TCA showed a significant decrease in contents of fatty acids, while TCA showed a stronger effect in decreasing fatty acid contents. Collectively, we suggest that HCD decreases lamb fatty acid contents by regulating bile acid composition.

## Highlights

• High-concentrate diet affects the metabolites composition of mutton.
• Both CA and TCA inhibit the accumulation of fatty acids in skeletal muscle cells.
• High-concentrate diet decreases mutton fatty acid contents by regulating bile acid composition.

## 1 Introduction

Currently, feeding sheep with high-concentrate diet has emerged as a sophisticated production technique in contrast to conventional grass feeding, facilitating an increase in the sales rate of livestock and thereby increasing overall production and profitability (Perez-Trejo et al., 2022). High-concentrate diet is a kind of diet that contains a higher level of easily fermentable carbohydrates and starch, and a lower level of fiber. By increasing the nutrient level of diet, this production strategy not only can significantly improve slaughter performance but also change the meat quality of livestock (Ma et al., 2023), which might be resulted from growth rate changes (Koch et al., 2023). A major effect of high concentrate diet to meat is that it significantly decreases the slice shear force of meat (Del Campo et al., 2021). While it has been reported that high-concentrate diet affects the sensory quality of mutton (Gao et al., 2014). The compounds influencing meat flavor and taste, including lipids, hydrophilic metabolites, and volatile compounds, are highly associated with meat quality and customers' choice (Wang et al., 2022).

Bile acids are not only amphipathic molecules that facilitate digestion, but also important physiological agents. It remains unclear whether bile acids have influence on lamb. Bile acids, cholesterol-derived endogenous metabolites, have a vital role in maintaining energy homeostasis. The process of absorption and digestion of dietary lipids is facilitated by bile acids (Zhong et al., 2022). Primary bile acids are those initially transformed from cholesterol in hepatocytes via two different metabolic pathways, including a classical pathway depending on cholesterol 7α-hydroxylase and an alternative pathway depending on sterol 27-hydroxylase (Jia et al., 2021). These bile acids are then conjugated with taurine or glycine, secreted into the gallbladder, and released into the intestinal lumen after food ingestion. The intestinal microbiota facilitates the biotransformation of bile acids from primary to secondary within the intestine (Yang, Yang, et al., 2023). The bile acids are then reabsorbed into enterohepatic circulation by the gut epithelium when they reach the distal part of the small intestine. During this process, bile acids can overflow into the systemic circulation and function as signaling molecules that influence almost all organs through the activation of dedicated bile acid receptors (Perino & Schoonjans, 2022).

Recently, metabolomics has been widely applied in analyzing the effects of pre-slaughter factors on meat quality. Metabolomics represents the downstream information of genomes, transcriptomes, and proteomes, which provides an overview of all small-molecule metabolites of living organisms (Creydt & Fischer, 2022a). This promotes researchers to compare the meat samples processed with diverse treatments and identify quality markers through metabolomics (Ramanathan et al., 2023), in which untargeted metabolomics using UHPLC-MS/MS platforms has become an increasingly important shotgun approach to analyze the chemistry of meat quality change (Creydt & Fischer, 2022b). While, untargeted metabolomics is usually combined with targeted metabolomics in order to provide more detailed information of the meat under different feeding strategies. In this study, the differential metabolites between control and HCD lamb were significantly enriched in primary bile acid biosynthesis. Thus, we hypothesized that bile acids were involved in high concentrate diet caused lamb lipid contents change. Furthermore, we have verified and quantified the bile acids contents. Given the central role of bile acids in fatty acid biosynthesis, we have profiled fatty acids and validated the effect of bile acids by in vitro study.

## 2 Material and methods

### 2.1 Animals

Ten male Hu Sheep were divided randomly into two groups at the age of day 60, including an HCD and control group in a commercial farm (Zhejiang Yihui Ecological Agriculture Co., Ltd., Zhejiang Province, China). All the sheep were fed with oat grass and peanut vine as roughage, while a pellet was used as concentrated feed with 22.24 % crude protein (Table S1). The diet for the sheep in the control group was comprised of 20 % of the pellet on dry matter basis, while the diet for the HCD group was comprised of 50 % pellet. Sheep were slaughtered at 9 months of age. The information of animal characteristics was shown in Table S2. Slaughtering was carried out in a commercial abattoir (Huzhou Changxing Muyuan Food Co., Ltd) by the slaughter house staff, the method of slaughtering followed the traditional procedures including stunning. The Institutional Animal Care and Use Committees at the Zhejiang Academy of Agricultural Sciences provided their approval to the experimental protocols used in animal research.

### 2.2 Sample preparation for UHPLC-MS/MS

Longissimus dorsi samples from thirteenth rib were collected and separately grounded with liquid nitrogen followed by the reconstitution of the resulting homogenate in methanol (MeOH). After vortex mixing, the samples were subjected to incubation for 5 min followed by centrifugation (15,000*g*, 20 min, 4 °C), after which the supernatant was diluted to 53 % MeOH. After that, the samples were centrifuged for the duration of 20 min at 15000 g, 4 °C before being incorporated into the LC-MS/MS system.

To examine bile acid, 100 mg of lamb sample were resuspended with liquid nitrogen followed by the incorporation of 0.9 mL of ddH_2_O. Following dilution, it was combined with a mixture of acetonitrile (ACN) and MeOH (8:2) containing mixed internal standards (IS). Samples were centrifuged (12,000 rpm, 20 min) and the supernatants were dried with a nitrogen blower followed by reconstitution with 100 μL of water/ACN (8:2) that contained formic acid (FA, 0.1 %). For fatty acid measurement, homogenization of 100 mg of the lamb samples was performed with 300 μL of mixed IS containing Isopropanol/ACN (1:1), and centrifuged for 10 min at 12,000 rpm.

### 2.3 UHPLC-MS/MS analysis

UHPLC-MS/MS was performed using a Vanquish UHPLC system (Thermo Fisher, USA) coupled with an Orbitrap Q Exactive TMHF mass spectrometer (Thermo Fisher) in Novogene Co., Ltd. (Beijing, China). Samples were applied to a Hypesil Gold column and eluted with a linear gradient. FA (1 %) in the mixture of water and MeOH were utilized as eluents in the positive polarity mode while the negative polarity mode's eluents were based on ammonium acetate and MeOH. A Q Exactive TM HF mass spectrometer was used.

### 2.4 Data processing and metabolite identification

Compound Discoverer 3.1 (CD3.1, ThermoFisher) was used to carry out peak picking, peak alignment, and quantification of individual metabolites in the UHPLC-MS/MS-generated raw data files. After that, the total spectral intensity was used to normalize the peak intensities. Using the normalized data, the molecular formula was predicted considering the molecular ion peaks along with fragment and additive ions. The accurate as well as relative quantitative results were acquired by matching the peaks with Cloud (https://www.mzcloud.org/), mz Vault, and Mass List data bile acids. The statistical software R, Python, and CentOS were used to conduct the statistical studies. Annotation of metabolites was done with the KEGG (https://www.genome.jp/kegg/pathway.html) databases.

### 2.5 The measurement of bile acids and fatty acids

Six stable isotope-labeled standards as well as thirty-three of the bile acid standards, or fifty standards of fatty acids and five stable isotope-labeled standards were acquired from ZZ Standards Co., LTD. (Shanghai, China). LC-MS/MS was conducted by injecting 2 μL supernatant. For the purpose of separation, Waters ACQUITY UPLC BEH C18 column (2.1 × 100 mm, 1.7 μm) was used which was kept at 50 °C. At 0.30 mL/min flow rate, the mobile phase, a mixture of 0.1 % FA in water and ACN, was introduced for bile acid measurement, while 0.05 % FA in water and Isopropanol/ACN (1:1) was introduced for fatty acid measurement.

### 2.6 Cell isolation and culture

Lamb longissimus dorsi was collected from Hu sheep, washed with PBS buffer containing antibiotic. The connective tissue and fat were removed. Then, the lamb sample was cut into small pieces. Subsequently, 0.2 % collagenase IV was added and sample was digested in a water bath of 37 °C for 1 h. Then, 0.25 % trypsin was added to digest for 20 min. After digestion was terminated, the samples were resuspended, and filtered, centrifuged at 1000 r/min for 10 min. The primary cells of the lamb longissimus dorsi were isolated and purified by Percoll density gradient centrifugation after being suspended in complete culture medium for precipitation. Resuspend the cells and cultured in a 37 °C and 5 % CO_2_ incubator. The cells were incubated with either 2 μΜ CA or 1 μΜ TCA for 2 days before fatty acid profile.

### 2.7 Data analysis

Data are presented as means ± SEM unless otherwise indicated. Differences were determined with independent-samples *t*-tests using SPSS 20.0 software (IBM Corp., USA.).

## 3 Results

### 3.1 Multivariate statistical analysis of lamb metabolites

We analyzed the lamb samples by UHPLC-QTOF/MS in the positive (ESI+) and negative ion (ESI-) modes. The ESI- and ESI+ modes respectively detected a total of 797 and 519 metabolites. Then, the metabolites were annotated based on secondary mass spectrometry and database mapping (Fig. S1). PCA was performed to illustrate the differences between control and HCD samples. The analysis revealed that the first two main components described 68.81 % of the variability, corresponding to 13.73 % and 55.08 % of the total variance in the ESI-mode (Fig. S2A). While 66.18 % variability was observed for the first two primary components corresponding to 47.47 %, and 18.71 % of the total variance in ESI+ mode (Fig. S2B).

**Fig. 1:**
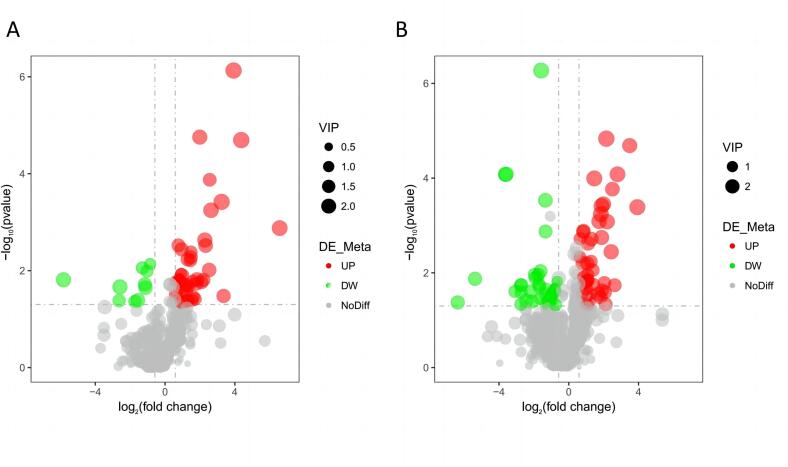
A volcano map showing ESI- (A) and ESI+ modes (B) of differential metabolites in lamb of high-concentrate diet.

PLS-DA was then performed to determine the lamb metabolic profile. PLS-DA analysis showed that the 70.68 % variability demonstrated by the first two primary components, represented total variances of 31.01 %, and 39.67 % in the ESI- mode (Fig. S1 A), while 67.42 % variability of the top two primary components accounted for variances of 44.99 %, and 22.43 % in ESI+ mode (Fig. S1B). A discernible metabolite profile was observed between HCD lamb and control, as evidenced by the separation and placement of samples in distinct quadrants. Due to the similar distribution of the score plot to that of PCA, and the results of validation tests of the PLS-DA model (Fig. S1C and D), we suggested that the validity of the profiling of metabolites distinguish the different groups. According to fold change >1.5 or < 0.67 and *p*<0.05, 52 and 83 differentially abundant metabolites were determined using ESI- (Fig. 1A, Table S3) and ESI+ (Fig. 1B, Table S4) mode, respectively, including 88 overabundant metabolites in HCD lamb and 47 excessively abundant metabolites in control lamb.

### 3.2 Bioinformatics analysis of differentially abundant metabolites

**Fig. 2:**
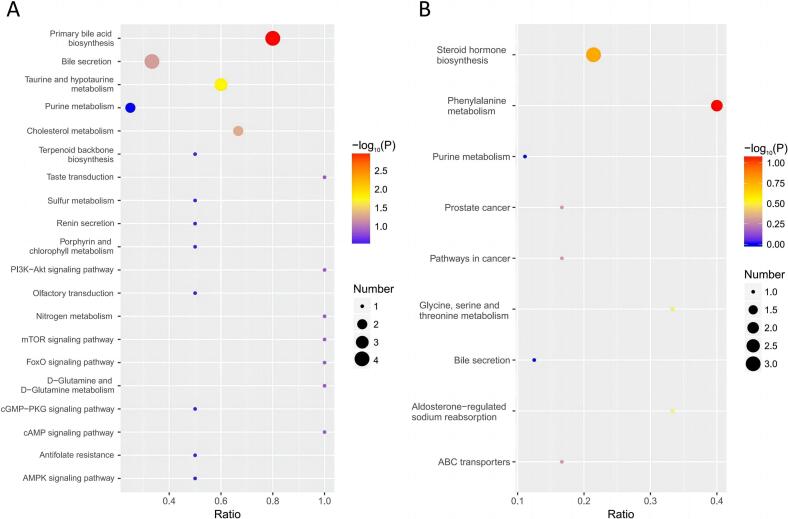
KEGG pathway enrichment analysis of differentially abundant metabolites in high-concentrate diet group in the ESI- (A) and ESI+ modes (B). Pathways are organized based on their corresponding *p*-value.

After differential metabolite identification, KEGG enrichment analysis was performed to reveal the effect of HCD on metabolic pathways. In ESI- mode, differentially abundant metabolites were significantly enriched in the biosynthesis of primary bile acid (map00120), metabolism of taurine and hypotaurine (map00430), and cholesterol metabolism (map04979)(Fig. 2A, Table S5). Differential metabolites in ESI+ mode were not significantly enriched, while Phenylalanine metabolism (map00360), Steroid hormone biosynthesis (map00140), Glycine, serine and threonine metabolism (map00260), Aldosterone-regulated sodium reabsorption (map04960) and ABC transporters (map02010) were among the top five enriched KEGG terms in positive mode (Fig. 2B, Table S6).

### 3.3 Differential bile acids in HCD lamb longissimus dorsi

In the KEGG terms that involved in bile acids, we found the content of taurochenodeoxycholic acid, taurine, TCA and L-glutamic acid significantly higher in HCD group, while the content of CA were significantly higher in control group (Table S3 and S4). Then, we reviewed the rest of differential bile acids and found the level of taurolithocholic acid sodium salt (TLCA) and taurochenodeoxycholic acid sodium salt (TCDCA) significantly higher in HCD lamb, while LCA and ursodeoxycholic acid (UDCA) were down-regulated (Table S3 and S4). These data indicated a diverse pattern of bile acids contents between HCD group and control group.

Thus, we were promoted to profile the bile acids composition between control and HCD lamb by non-targeted metabolomics. A total of fifteen bile acids were identified, among which seven bile acids differed markedly between the groups, including TDCA, TCDCA, CDCA, CA, CDCA-3Gln, LCA and TCA. These differential bile acids can be classified into four primary and three secondary, or four conjugated and three unconjugated bile acids. Similar to the non-targeted metabolomics results, the content of TDCA, TCDCA, and TCA were considerably lower in the control lamb (*p*<0.05), while considerably lower levels of LCA, CA, CDCA, and CDCA-3Gln were observed in HCD lamb (*p*<0.05) (Table 1).

**Table 1:** Bile acid profile of lamb longissimus dorsi between control and HCD groups ng/g.

name	Control	HCD
Taurodeoxycholic acid sodium salt (TDCA)	48.86 ± 12.61	159.23 ± 44.82*
Hyodeoxycholic acid (HDCA)	4.90 ± 1.45	2.69 ± 1.00
12-ketolithocholic acid (12- ketoLCA)	9.44 ± 1.98	7.98 ± 1.38
Deoxycholic acid (DCA)	217.41 ± 111.99	9.96 ± 3.51
Taurochenodeoxycholic acid sodium salt (TCDCA)	43.09 ± 17.60	130.25 ± 23.83*
Chenodeoxycholic acid (CDCA)	85.42 ± 27.12	1.62 ± 0.38*
Cholic acid (CA)	921.43 ± 442.43	9.90 ± 3.66*
Chenodeoxycholic acid-3-beta-D-glucuronide (CDCA-3Gln)	16.40 ± 3.55	0.76 ± 0.05*
Lithocholic acid (LCA)	19.67 ± 1.32	11.83 ± 1.34*
Glycochenodeoxycholic acid sodium salt (GCDCA)	13.68 ± 9.02	3.19 ± 1.41
Glycocholic acid hydrate (GCA)	48.96 ± 25.08	22.71 ± 6.03
Taurolithocholic acid sodium salt (TLCA)	3.79 ± 0.75	4.80 ± 0.92
Taurocholic acid (TCA)	166.93 ± 50.78	405.01 ± 96.99
Tauro-alpha-Muricholic acid sodium salt (T-α-MCA)	3.35 ± 1.28	1.90 ± 0.78
Glycodeoxycholic acid (GDCA)	7.50 ± 3.39	1.60 ± 0.42

Note: data are presented as MEAN ± SEM, *n* = 5, * means *p* < 0.05.

### 3.4 Differential fatty acids in the lamb longissimus dorsi between HCD and control groups

Overall, 48 fatty acids were found, with 28 of them showing significantly lower levels in the HCD group, including C15:0, C15:1, C15:1 T, C16:0, C16:1, C16:1 T, C17:0, C17:1, C17:1 T, C18:0, C18:1(n-9), C18:1(n-7), C18:1(n−12)T, C18:1(n-9)T, C18:1(n-7)T, C18:2(n-6), C18:2(n-6)T, C20:0, C22:1, C22:1 T, C20:3(n-3), C23:0, C22:2, C20:5, C22:5(n-3), C24:1 and C22:6 (*p*<0.05) (Table 2). The rest of fatty acids showed no significant difference between two groups.

**Table 2:** Fatty acids profile of lamb longissimus dorsi between control and high-concentrate diet groups ng/g.

Name	Control	HCD
Decanoic acid (C10:0)	402.80 ± 89.96	238.20 ± 34.07
Dodecanoic acid (C12:0)	391.66 ± 121.39	211.61 ± 22.78
Tridecanoic acid (C13:0)	27.50 ± 1.82	22.71 ± 1.33
Tetradecanoic acid (C14:0)	8004.59 ± 1876.64	4634.21 ± 456.74
Myristoleic acid (C14:1)	326.35 ± 89.04	160.40 ± 16.46
Myristelaidic acid (C14:1 T)	366.09 ± 164.38	58.29 ± 3.70
Pentadecanoic acid (C15:0)	1215.07 ± 173.52	582.53 ± 72.93*
cis-10-Pentadecenoic acid (C15:1)	31.00 ± 6.84	12.52 ± 1.83*
trans-10-Pentadecenoic acid (C15:1 T)	46.91 ± 6.57	31.09 ± 1.42*
Hexadecanoic acid (C16:0)	89,179.56 ± 7436.88	44,765.42 ± 4014.39*
Palmitoleic acid (C16:1)	15,941.07 ± 3261.84	8094.44 ± 937.73*
Palmitelaidic acid (C16:1 T)	6723.03 ± 343.78	4288.79 ± 669.86*
Heptadecanoic acid (C17:0)	6988.69 ± 912.21	2404.29 ± 234.37*
cis-10-Heptadecenoic acid (C17:1)	7535.43 ± 1506.77	3350.01 ± 538.05*
trans-10-Heptadecenoic acid (C17:1 T)	450.26 ± 85.49	197.60 ± 27.05*
Octadecanoic acid (C18:0)	52,259.17 ± 6226.20	25,032.25 ± 3907.08*
Petroselinic acid (C18:1(n-12))	85,121.34 ± 13,197.91	42,799.03 ± 6315.05*
Oleic acid (C18:1(n-9))	91,720.86 ± 14,719.47	45,726.71 ± 6729.29*
cis-Vaccenic acid (C18:1(n-7))	4395.72 ± 102.29	2910.32 ± 180.07*
Petroselaidic acid (C18:1(n-12)T)	12,661.20 ± 1543.11	2968.07 ± 473.28*
Elaidic acid (C18:1(n-9)T)	12,084.54 ± 1613.81	2945.28 ± 446.32*
trans-Vaccenic acid (C18:1(n-7)T)	169,563.70 ± 13,771.38	55,071.07 ± 4168.27*
Linoleic acid (C18:2(n-6))	7491.71 ± 897.95	2388.03 ± 190.97*
Linoelaidic acid (C18:2(n-6)T)	475.81 ± 91.16	227.99 ± 25.29*
trans-7-Nonadecenoic acid (C19:1(n-12)T)	494.10 ± 130.67	279.34 ± 71.65
trans-10-Nonadecenoic acid (C19:1(n-9)T)	2647.07 ± 588.09	1726.54 ± 257.08
Arachidic acid (C20:0)	7302.06 ± 1521.05	4020.02 ± 476.54*
gamma-Linolenic acid (C18:3(n-6))	2127.34 ± 493.63	2547.76 ± 510.08
cis-11-Eicosenoic acid (C20:1)	448.17 ± 143.86	309.15 ± 49.03
trans-11-Eicosenoic acid (C20:1 T)	6883.02 ± 1438.47	4077.39 ± 501.58
alpha-Linolenic acid (C18:3(n-3))	732.04 ± 242.46	385.40 ± 55.76
Heneicosanoic acid (C21:0)	3113.61 ± 705.18	2475.00 ± 404.94
cis-11,14-Eicosadienoic acid (C20:2)	1435.03 ± 249.36	1313.74 ± 215.41
Docosanoic acid (C22:0)	528.19 ± 146.47	422.66 ± 81.14
homo-gamma-Linolenic acid (C20:3(n-6))	436.96 ± 110.13	566.78 ± 97.10
Erucic acid (C22:1)	4204.35 ± 495.51	1699.15 ± 237.63*
Brassidic acid (C22:1 T)	120,981.1 ± 10,988.27	55,670.06 ± 7142.89*
cis-11,14,17-Eicosatrienoic acid (C20:3(n-3))	136.17 ± 28.47	60.23 ± 10.23*
Arachidonic acid (C20:4)	279.89 ± 82.08	317.37 ± 63.20
Tricosanoic acid (C23:0)	4612.67 ± 534.99	1148.38 ± 77.37*
cis-13,16-Docosadienoic acid (C22:2)	6689.10 ± 1093.51	3510.85 ± 623.22 *
cis-5,8,11,14,17-Eicosapentaenoic acid (C20:5)	10,979.60 ± 947.77	3018.93 ± 370.26*
cis-7,10,13,16-Docosic acidtraenoic acid (C22:4)	2967.07 ± 434.73	2157.89 ± 384.96
cis-7,10,13,16,19-Docosapentaenoic acid (C22:5(n-3))	154.84 ± 31.30	70.81 ± 14.14*
cis-4,7,10,13,16-Docosapentaenoic acid (C22:5(n-6))	2931.79 ± 807.46	3855.68 ± 811.28
Tetracosanoic acid (C24:0)	271,356.42 ± 53,249.25	154,247.47 ± 26,563.53
Nervonic acid (C24:1)	3463.27 ± 775.98	1380.45 ± 148.42*
cis-4,7,10,13,16,19-Docosahexaenoic acid (C22:6)	1397.83 ± 378.75	432.01 ± 45.56*

Note: data are presented as MEAN ± SEM, n = 5, * means *p* < 0.05.

### 3.5 Differential fatty acids in the longissimus dorsi cells following CA or TCA treatment

When compared with control group, we found the content of C11:0, C12:0, C13:0, C14:0, C14:1, C14:1 T, C15:0, C15:1, C16:0, C16:1, C16:1 T, C17:0, C17:1 T, C18:1(n-12), C18:1(n-9), C18:1(n-7), C18:1(n-12)T, C18:1(n-7)T, C18:2(n-6), C19:1(n-12)T, C19:1(n-9)T, C20:0, C20:1, C20:1 T, C21:0, C20:2, C22:1, C20:4, C23:0, C22:2, C20:5, C22:4, C22:5(n-3), C24:1, C22:6 significantly lower in CA treated cells, while the content of C10:0, C18:0, C18:1(n-9)T, C22:0, C22:1 T, C22:5(n-6) and C24:0 were significant higher in CA treatment group (*p*<0.05) (Table 3).

**Table 3:** Fatty acids profile of sheep skeletal muscle cells treated with CA and TCA ng/g.

Name	Control	CA	TCA
C10:0	0.50 ± 0.01^c^	1.02 ± 0.04^b^	1.37 ± 0.05^a^
C11:0	3.15 ± 0.22^a^	1.25 ± 0.06^b^	2.55 ± 0.04^a^
C12:0	55.91 ± 1.18^a^	46.61 ± 0.92^b^	46.11 ± 0.13^b^
C13:0	16.54 ± 0.32^a^	10.43 ± 0.26^b^	11.87 ± 0.48^b^
C14:0	167.60 ± 0.90^a^	136.00 ± 1.55^b^	136.06 ± 1.84^b^
C14:1	28.93 ± 0.63^a^	17.67 ± 0.37^b^	17.34 ± 0.71^b^
C14:1 T	30.75 ± 0.86^a^	19.32 ± 0.69^b^	21.27 ± 0.88^b^
C15:0	187.09 ± 2.41^a^	144.49 ± 1.99^b^	113.82 ± 2.15^c^
C15:1	30.92 ± 0.56^a^	26.75 ± 0.41^b^	30.11 ± 0.41^a^
C16:0	3138.44 ± 7.81^a^	2673.45 ± 17.02^b^	2479.69 ± 8.78^c^
C16:1	314.88 ± 2.63^a^	227.75 ± 2.13^b^	209.42 ± 2.64^c^
C16:1 T	341.26 ± 2.63^a^	276.81 ± 3.98^b^	332.81 ± 1.82^a^
C17:0	117.49 ± 1.45^a^	95.81 ± 1.40^b^	84.13 ± 1.02^c^
C17:1	201.04 ± 1.05^a^	198.43 ± 1.59^a^	143.74 ± 2.31^b^
C17:1 T	178.04 ± 2.73^a^	108.58 ± 4.17^b^	96.50 ± 3.35^b^
C18:0	1978.59 ± 11.95^c^	2662.67 ± 24.83^a^	2236.42 ± 16.09^b^
C18:1(n-12)	3534.37 ± 17.66^a^	3112.93 ± 28.06^b^	2375.57 ± 14.70^c^
C18:1(n-9)	3222.30 ± 9.34^a^	2935.29 ± 31.81^b^	2177.09 ± 36.17^c^
C18:1(n-7)	64.53 ± 0.84^a^	44.07 ± 1.83^b^	42.81 ± 1.37^b^
C18:1(n-12)T	56.58 ± 0.41^a^	46.56 ± 1.86^b^	23.96 ± 1.63^c^
C18:1(n-9)T	161.81 ± 6.52^c^	364.16 ± 9.27^a^	222.38 ± 4.55^b^
C18:1(n-7)T	2667.76 ± 10.79^a^	1807.18 ± 13.61^b^	1251.21 ± 19.68^c^
C18:2(n-6)	445.06 ± 3.61^a^	316.53 ± 7.60^b^	282.39 ± 5.47^c^
C19:1(n-12)T	40.49 ± 1.20^a^	24.21 ± 0.59^b^	16.30 ± 0.36^c^
C19:1(n-9)T	235.23 ± 3.87^a^	156.74 ± 2.06^b^	163.27 ± 2.68^b^
C20:0	452.20 ± 3.01^a^	236.39 ± 3.88^b^	181.82 ± 2.39^c^
C18:3(n-6)	379.23 ± 1.95^a^	387.12 ± 3.12^a^	321.16 ± 4.29^b^
C20:1	221.64 ± 2.80^a^	54.69 ± 1.98^c^	125.86 ± 3.21^b^
C20:1 T	408.16 ± 3.15^a^	258.51 ± 4.45^b^	109.69 ± 3.56^c^
C18:3(n-3)	12.97 ± 0.26^a^	12.89 ± 0.41^a^	8.56 ± 0.27^b^
C21:0	457.68 ± 3.02^a^	393.98 ± 6.79^b^	251.41 ± 3.35^c^
C20:2	145.65 ± 1.93^a^	130.16 ± 1.49^b^	88.71 ± 1.50^c^
C22:0	103.63 ± 1.30^b^	114.00 ± 1.63^a^	82.64 ± 1.32^c^
C22:1	802.10 ± 5.28^a^	719.07 ± 7.21^b^	439.85 ± 6.95^c^
C22:1 T	5010.25 ± 19.43^b^	5437.07 ± 7.08^a^	3981.74 ± 8.15^c^
C20:3(n-3)	4.28 ± 0.12^b^	3.85 ± 0.15^b^	4.99 ± 0.13^a^
C20:4	200.49 ± 1.65^a^	187.94 ± 1.26^b^	132.58 ± 1.75^c^
C23:0	917.95 ± 4.25^a^	878.10 ± 3.90^b^	598.14 ± 4.88^c^
C22:2	1908.57 ± 6.02^a^	1535.23 ± 6.83^b^	1385.66 ± 7.71^c^
C20:5	385.78 ± 3.71^a^	260.23 ± 3.90^b^	238.30 ± 4.17^c^
C22:4	315.92 ± 3.84^a^	259.76 ± 2.88^b^	213.02 ± 2.66^c^
C22:5(n-3)	14.42 ± 0.32^a^	12.95 ± 0.20^b^	15.03 ± 0.38^a^
C22:5(n-6)	122.00 ± 3.46^b^	136.54 ± 2.54^a^	113.44 ± 2.28^b^
C24:0	2780.30 ± 7.22^b^	3026.61 ± 16.40^a^	2372.92 ± 19.05^c^
C24:1	1555.23 ± 12.18^a^	1388.60 ± 17.62^b^	830.48 ± 18.54^c^
C22:6	28.28 ± 1.52^a^	16.43 ± 0.69^b^	14.23 ± 0.39^c^

Note: data are presented as MEAN ± SEM, *n* = 3 repeats, * means *p* < 0.05.

Comparing with control group, the contents of C11:0, C12:0, C13:0, C14:0, C14:1, C14:1 T, C15:0, C16:0, C16:1, C17:0, C17:1, C17:1 T, C18:1(n-12), C18:1(n-9), C18:1(n-7), C18:1(n-12)T, C18:1(n-7)T, C18:2(n-6), C19:1(n-12)T, C19:1(n-9)T, C20:0, C20:1, C20:1 T, C18:3(n-3), C21:0, C20:2, C22:0, C22:1, C22:1 T, C20:4, C23:0, C22:2, C20:5, C22:4, C24:0, C24:1, C22:6 were significantly lower in TCA treated cells, while the content of C10:0, C18:0, C18:1(n-9)T, C20:3(n-3) were significant higher (*p*<0.05) (Table 3).

When comparing with CA treated cells, the contents of C15:0, C16:0, C16:1, C17:0, C17:1, C18:0, C18:1(n-12), C18:1(n-9), C18:1(n-12)T, C18:1(n-9)T, C18:1(n-7)T, C18:2(n-6), C19:1(n-12)T, C20:0, C18:3(n-6), C20:1 T, C18:3(n-3), C21:0, C20:2, C22:0, C22:1, C22:1 T, C20:4, C23:0, C22:2, C20:5, C22:4, C22:5(n-6), C24:0, C24:1 and C22:6 were significantly lower in TCA treated cells, while the content of C10:0, C11:0, C15:1, C16:1 T, C20:1, C20:3(n-3) and C22:5(n-3) were significantly higher in TCA treated cells when comparing with CA treated cells (*p*<0.05) (Table 3).

## 4 Discussion

The intramuscular content of fatty acid and its composition are important indicators of meat quality, which also play essential roles in metabolic homeostasis (Valdes-Hernandez et al., 2023). In this study, we have found that high-concentrate diet changed the metabolite composition of lamb. Bile acids played an essential role in regulating fatty acid composition. Both CA and TCA decrease the contents of various species of fatty acids. These results highlight an attractive characteristic of bile acid in regulating lamb fatty acid accumulation.

Recently, bile acids have emerged as important regulators of various physiological and pathological processes. Diet nutrition is known to influence bile acid secretion, however, current understanding to the molecular species of bile acid remains unclear (Yoshitsugu et al., 2019). In mice, it has been reported that high-fat diet changes gut microbiota composition and metabolism, which, in turns, influences bile acid composition (Ocvirk & O'Keefe, 2021). In pig, it has been reported that dietary fatty acids changed the bile acid composition of liver, plasma, and colon (Manjarin et al., 2022). Similar to previous studies, we found the composition of bile acid in lamb changed after fed with high concentrate diet, while Metabolomics analysis also showed that biosynthesis of primary bile acid, metabolism of taurine and hypotaurine, and cholesterol metabolism were key KEGG pathways that high concentrate diet influences lamb. Since cholesterol is a precursor of bile acids, these evidences indicate that bile acids are important regulator in lamb fed with high concentrate diet.

Since FXR was reported low expression in skeletal muscle (Perino & Schoonjans, 2022), TGR5 and S1PR2 are considered the key receptors that interacts with bile acids in skeletal muscle (Kitada et al., 2016; Maldonado et al., 2023). TGR5 is highly expressed in the muscle, adipose and intestine, which participates in the stimulation energy metabolism. Bile acids can induce type 2 iodothyronine deiodinase expression in skeletal muscle cell via a TGR5-dependent manner (Watanabe et al., 2006). D2 then induces uncoupling protein expression by converting thyroxine to tri-iodothyronine, which finally dissipate the proton gradient in electron transport chain and influence energy homeostasis (Fujiwara et al., 2023). Thus, activation of TGR5 by bile acids or its agonist inhibits fatty acid uptake and decreases lipid accumulation (Wang et al., 2024). S1PR2 is the receptor for bile acids conjugated with taurine including TCA, TDCA and TUDCA (Yang, Yu, et al., 2023). The conjugation of taurine with bile acids increase the polarity of bile acids, which facilitates the absorption of lipid and lipid-solved vitamins, and reduces the cytotoxicity of hydrophobic bile acids (Vettorazzi et al., 2016). It has been reported that high-fat diet triggers the production bile acids conjugated taurine in the bile acid pool (Devkota et al., 2012). Activation of S1PR2 by taurine-conjugated bile acids increases sphingosine kinase expression, which promotes lipid metabolism (Studer et al., 2012). In this study, we found the content of TDCA, TCDCA, and TCA increased in HCD lamb, while the content of LCA, CA, CDCA, and CDCA-3Gln decreased in HCD lamb. Furthermore, the content of plenty species of fatty acids decreased. These evidences indicated that HCD decrease fatty acid accumulation in lamb by changing the composition of bile acid.

Our in vitro study showed that both CA and TCA decreased the content of various species of fatty acids, in which more species of fatty acids showed relative lower contents in the cells treated with TCA. Taurine is not only a free osmolyte, but also a conjugated metabolite, which can modulate energy and lipid metabolism through regulating proteins involves mitochondrial biogenesis and respiratory function (Murakami, 2015). Supplying taurine in diet increases fatty acid oxidation (De Carvalho et al., 2021). Previous research studies have also found that Taurine-conjugated UDCA could reduce fat deposition in hepatocytes (Cui et al., 2023). TUDCA increased oxidation of fatty acids via AMPK/HSL signaling pathway in brown fat tissue (Dos Reis Araujo et al., 2022). A study on fish also showed that high lipid diet supplemented with TCA decreased lipid deposition in fish (Xu et al., 2022). These data indicated that taurine-conjugated bile acids exhibited a stronger inhibitory effect to the accumulation of various species of fatty acid in lamb.

## 5 Conclusions

Collectively, the high concentrate diet influences lamb metabolites. Our results revealed that high concentrate diet changed the bile acid composition by increasing the content of TCA, TCDCA, and TDCA, and decreasing the content of CA, LCA, CDCA, and CDCA-3Gln in lamb. This composition changes of bile acids resulted in the decrease of fatty acids accumulation in lamb longissimus dorsi. Our data also indicated that taurine-conjugated bile acids exhibited a stronger inhibitory effect to fatty acid accumulation in lamb.

## CRediT authorship contribution statement

**Kaizhi Zheng:** Writing – review & editing, Writing – original draft, Investigation. **Liangyong Guo:** Data curation, Conceptualization. **Yang Cao:** Data curation, Conceptualization. **Yuyang Yin:** Methodology, Investigation. **Hui Gao:** Methodology, Investigation. **Xiaowei Zhang:** Methodology, Investigation. **Junfang Jiang:** Investigation. **Jinbing Li:** Investigation. **Xin Huang:** Funding acquisition. **Kui Li:** Supervision. **Sangang He:** Project administration.

## Declaration of competing interest

The authors declare that they have no known competing financial interests or personal relationships that could have appeared to influence the work reported in this paper.

## Data Availability

Metabolomics data have been deposited to the EMBL-EBI MetaboLights database (DOI: 10.1093/nar/gkad1045, PMID:37971328) with the identifier MTBLS10322.

## Appendix A Supplementary data

Table S1. Nutrition composition of the pellet. Table S2. Animal characteristics information. Table S3. Differentially abundant compounds of mutton longissimus dorsi between control and high-concentrate diet groups in negative ion mode. Table S4. Differentially abundant compounds of mutton longissimus dorsi between control and high-concentrate diet groups in positive ion mode. Table S5. The KEGG functional enrichments of differential metabolites in negative ion mode. Table S6. The KEGG functional enrichments of differential metabolites in positive ion mode. Figure S1. KEGG annotation classification of differentially abundant metabolites in HCD mutton in the ESI- (A) and ESI+ modes (B). Figure S2. PCA scores plot of control and HCD mutton in the ESI- (A) and ESI+ modes (B). Image 1

## References

[bb0005] del Campo M., Manteca X., Soares de Lima J.M., Brito G., Hernandez P., Sanudo C., Montossi F. (2021). Effect of different finishing strategies and steer temperament on animal welfare and instrumental meat tenderness. Animals (Basel).

[bb0010] de Carvalho F.G., Brandao C.F.C., Batitucci G., Souza A.O., Ferrari G.D., Alberici L.C., de Freitas E.C. (2021). Taurine supplementation associated with exercise increases mitochondrial activity and fatty acid oxidation gene expression in the subcutaneous white adipose tissue of obese women. Clinical Nutrition.

[bb0015] Creydt M., Fischer M. (2022). Food authentication: Truffle species classification by non-targeted lipidomics analyses using mass spectrometry assisted by ion mobility separation. Molecular Omics.

[bb0020] Creydt M., Fischer M. (2022). Food metabolomics: Latest hardware-developments for nontargeted food authenticity and food safety testing. Electrophoresis.

[bb0025] Cui N., Zhang W., Su F., Zhang Z., Qiao W., Sun Y., Wang Q. (2023). Metabolomics and lipidomics study unveils the impact of tauroursodeoxycholic acid on hyperlipidemic mice. Molecules.

[bb0030] Devkota S., Wang Y., Musch M.W., Leone V., Fehlner-Peach H., Nadimpalli A., Chang E.B. (2012). Dietary-fat-induced taurocholic acid promotes pathobiont expansion and colitis in Il10−/− mice. Nature.

[bb0035] Dos Reis Araujo T., Roberta Rodrigues Muniz M., Lourenconi Alves B., dos Monali Barreto Santos L., Fernandes Bonfim M., Alves da Silva Junior J., Magalhaes Carneiro E. (2022). Tauroursodeoxycholic acid improves glucose tolerance and reduces adiposity in normal protein and malnourished mice fed a high-fat diet. Food Research International.

[bb0040] Fujiwara Y., Miyasaka Y., Ninomiya A., Miyazaki W., Iwasaki T., Ariyani W., Koibuchi N. (2023). Effects of perfluorooctane sulfonate on cerebellar cells via inhibition of type 2 iodothyronine deiodinase activity. International Journal of Molecular Sciences.

[bb0045] Gao X., Wang Z., Miao J., Xie L., Dai Y., Li X., Dai R. (2014). Influence of different production strategies on the stability of color, oxygen consumption and metmyoglobin reducing activity of meat from ningxia tan sheep. Meat Science.

[bb0050] Jia W., Wei M., Rajani C., Zheng X. (2021). Targeting the alternative bile acid synthetic pathway for metabolic diseases. Protein & Cell.

[bb0055] Kitada Y., Kajita K., Taguchi K., Mori I., Yamauchi M., Ikeda T., Morita H. (2016). Blockade of sphingosine 1-phosphate receptor 2 signaling attenuates high-fat diet-induced adipocyte hypertrophy and systemic glucose intolerance in mice. Endocrinology.

[bb0060] Koch C., Schonleben M., Mentschel J., Gores N., Fissore P., Cohrs I., Ghaffari M.H. (2023). Growth performance and economic impact of simmental fattening bulls fed dry or corn silage-based total mixed rations. Animal.

[bb0065] Ma X., Guo X., La Y., Wu X., Chu M., Bao P., Liang C. (2023). Integrative analysis of proteomics and transcriptomics of longissimus dorsi with different feeding systems in yaks. Foods.

[bb0070] Maldonado L., Orozco-Aguilar J., Valero-Breton M., Tacchi F., Cifuentes-Silva E., Cabello-Verrugio C. (2023). Differential fibrotic response of muscle fibroblasts, myoblasts, and myotubes to cholic and deoxycholic acids. Advances in Experimental Medicine and Biology.

[bb0075] Manjarin R., Dillard K., Coffin M., Hernandez G.V., Smith V.A., Noland-Lidell T., Maj M. (2022). Dietary fat composition shapes bile acid metabolism and severity of liver injury in a pig model of pediatric NAFLD. American Journal of Physiology. Endocrinology and Metabolism.

[bb0080] Murakami S. (2015). Role of taurine in the pathogenesis of obesity. Molecular Nutrition & Food Research.

[bb0085] Ocvirk S., O’Keefe S.J.D. (2021). Dietary fat, bile acid metabolism and colorectal cancer. Seminars in Cancer Biology.

[bb0090] Perez-Trejo E., Andrade-Montemayor H.M., Robles-Jimenez L.E., Humaran M., Orozco-Estrada E., Hernandez-Hernandez J., Gonzalez-Ronquillo M. (2022). Effect of replacing soybean meal (Glycine max) with sesame meal (Sesamum indicum) on productive traits, carcass characteristics, and gross profit margin in fattening lamb’s diets. Tropical Animal Health and Production.

[bb0095] Perino A., Schoonjans K. (2022). Metabolic messengers: Bile acids. Nature Metabolism.

[bb0100] Ramanathan R., Kiyimba F., Suman S.P., Mafi G.G. (2023). The potential of metabolomics in meat science: Current applications, trends, and challenges. Journal of Proteomics.

[bb0105] Studer E., Zhou X., Zhao R., Wang Y., Takabe K., Nagahashi M., Hylemon P.B. (2012). Conjugated bile acids activate the sphingosine-1-phosphate receptor 2 in primary rodent hepatocytes. Hepatology.

[bb0110] Valdes-Hernandez J., Ramayo-Caldas Y., Passols M., Sebastia C., Criado-Mesas L., Crespo-Piazuelo D., Folch J.M. (2023). Global analysis of the association between pig muscle fatty acid composition and gene expression using RNA-Seq. Scientific Reports.

[bb0115] Vettorazzi J.F., Ribeiro R.A., Borck P.C., Branco R.C., Soriano S., Merino B., Carneiro E.M. (2016). The bile acid TUDCA increases glucose-induced insulin secretion via the cAMP/PKA pathway in pancreatic beta cells. Metabolism.

[bb0120] Wang B., Zhao X., Zhang B., Cui Y., Nueraihemaiti M., Kou Q., Luo H. (2022). Assessment of components related to flavor and taste in tan-lamb meat under different silage-feeding regimens using integrative metabolomics. Food Chemistry: X.

[bb0125] Wang H., Wang J., Cui H., Fan C., Xue Y., Liu H., Xu M. (2024). Inhibition of fatty acid uptake by TGR5 prevents diabetic cardiomyopathy. Nature Metabolism.

[bb0130] Watanabe M., Houten S.M., Mataki C., Christoffolete M.A., Kim B.W., Sato H., Auwerx J. (2006). Bile acids induce energy expenditure by promoting intracellular thyroid hormone activation. Nature.

[bb0135] Xu J., Li X., Yao X., Xie S., Chi S., Zhang S., Tan B. (2022). Protective effects of bile acids against hepatic lipid accumulation in hybrid grouper fed a high-lipid diet. Frontiers in Nutrition.

[bb0140] Yang C., Yang L., Yang Y., Wan M., Xu D., Pan D., Sun G. (2023). Effects of flaxseed powder in improving non-alcoholic fatty liver by regulating gut microbiota-bile acids metabolic pathway through FXR/TGR5 mediating. Biomedicine & Pharmacotherapy.

[bb0145] Yang R., Yu W., Lin L., Jin M., Hu S., Jiang B., Lu E. (2023). Profiling of bile acids and activated receptor S1PR2 in gingival tissues of periodontitis patients. Journal of Periodontology.

[bb0150] Yoshitsugu R., Kikuchi K., Iwaya H., Fujii N., Hori S., Lee D.G., Ishizuka S. (2019). Alteration of bile acid metabolism by a high-fat diet is associated with plasma transaminase activities and glucose intolerance in rats. Journal of Nutritional Science and Vitaminology (Tokyo).

[bb0155] Zhong W.L., Wang H., Yang Y., Zhang Y.L., Lai H.J., Cheng Y.L., Zhai Q.W. (2022). High-protein diet prevents fat mass increase after dieting by counteracting-enhanced lipid absorption. Nature Metabolism.

