# Supplementary material for: High-concentrate diet decreases lamb fatty acid contents by regulating bile acid composition
Source: Food Chem X. 2024 Oct 5;24:101871. doi: 10.1016/j.fochx.2024.101871 (PMC11838137; doi:10.1016/j.fochx.2024.101871)
Supplement: Supplementary file 1 — Table S1. Nutrition composition of the pellet. Table S2. Animal characteristics information. Table S3. Differentially abundant compounds of mutton longissimus dorsi between control and high-concentrate diet groups in negative ion mode. Table S4. Differentially abundant compounds of mutton longissimus dorsi between control and high-concentrate diet groups in positive ion mode. Table S5. The KEGG functional enrichments of differential metabolites in negative ion mode. Table S6. The KEGG functional enrichments of differential metabolites in positive ion mode. Figure S1. KEGG annotation classification of differentially abundant metabolites in HCD mutton in the ESI- (A) and ESI+ modes (B). Figure S2. PCA scores plot of control and HCD mutton in the ESI- (A) and ESI+ modes (B). [file mmc1.docx]

**Supplemental materials**

**High-concentrate diet decreases mutton fatty acid contents by regulating bile acid composition**

Kaizhi Zheng^a^, Liangyong Guo^c^, Yang Cao^a^, Yuyang Yin^c^, Hui Gao^b^, Xiaowei Zhang^b^, Junfang Jiang^a^, Jinbing Li^d^, Xin Huang^a*^, Kui Li^b*^, and Sangang He^a*^

^a^Institute of Animal Husbandry and Veterinary, Zhejiang Academy of Agricultural Sciences, Hangzhou 310021, China.

^b^Animal Husbandry Technology Promotion and Breeding Livestock and Poultry Monitoring Station of Zhejiang Province, Hangzhou 310000, China.

^c^Huzhou Academy of Agricultural Sciences, Huzhou 313000, China.

Shangyu District Animal Husbandry and Veterinary Technology Promotion Center, Shaoxing 312300, China.

*Correspondence and requests for materials should be addressed to Xin Huang (hxin500@163.com, (0571)86404123) Kui Li [(kuili76@yeah.net,](mailto:(kuili76@yeah.net,) (0571)86404123)，and Sangang He [(hesangang3@163.com,](mailto:(jiangjunfang1031@sina.com,) (0571)86404123) .

**Table S1. Nutrition composition of the pellet**

| Item | Content (%) |
| --- | --- |
| Dry matter | 88.60 |
| Crude fat | 3.01 |
| Crude protein | 22.24 |
| Crude fiber | 5.58 |
| Acid detergent fiber | 9.90 |
| Neutral detergent fiber | 21.80 |
| Ca | 0.76 |
| P | 0.58 |
| Ash | 6.90 |

**Table S2.** Animal characteristics information

| Group | Initial weight (kg) | Final weight (kg) | Average daily gain (g) | Carcass weight (kg) |
| --- | --- | --- | --- | --- |
| Control | 19.58±1.20 | 49.36±0.94 | 141.81±7.46 | 24.81±0.35 |
| HCD | 19.62±0.71 | 61.27±1.15* | 198.33±4.02* | 31.25±0.39* |

Note: data are presented as MEAN±SEM, n=5, * means p<0.05.

**Table S3.** Differentially abundant compounds of mutton longissimus dorsi between control and high-concentrate diet groups in negative ion mode.

| Compound ID | Compound name | Formula | Trend | Fold change | P-value |
| --- | --- | --- | --- | --- | --- |
| Com_7069_neg | N-(1,3-benzodioxol-5-yl)-7-chloroquinolin-4-amine | C16 H11 Cl N2 O2 | up | 15.23 | 0.0000 |
| Com_5370_neg | Catechol | C6 H6 O2 | up | 3.98 | 0.0000 |
| Com_9224_neg | 2,4-Dihydroxybenzoic acid | C7 H6 O4 | up | 20.66 | 0.0000 |
| Com_5013_neg | LPG 20:0 | C26 H53 O9 P | up | 5.90 | 0.0001 |
| Com_5836_neg | 7-Hydroxy-3,4-dihydrocarbostyril | C9 H9 N O2 | up | 9.46 | 0.0004 |
| Com_11642_neg | Cinnamoylglycine | C11 H11 N O3 | up | 6.21 | 0.0006 |
| Com_4183_neg | 3-(methylsulfanyl)-5H-[1,2,4]triazino[5,6-b]indole | C10 H8 N4 S | up | 95.12 | 0.0013 |
| Com_12330_neg | 3-Indoxyl sulphate | C8 H7 N O4 S | up | 4.86 | 0.0023 |
| Com_6097_neg | LPG 16:1 | C22 H43 O9 P | up | 1.70 | 0.0030 |
| Com_1025_neg | PE (14:0e/18:1) | C37 H74 N O7 P | up | 5.01 | 0.0030 |
| Com_483_neg | L-Glutamic acid | C5 H9 N O4 | up | 1.93 | 0.0036 |
| Com_2375_neg | LPS 20:3 | C26 H46 N O9 P | up | 2.80 | 0.0042 |
| Com_2583_neg | LPS 19:1 | C25 H48 N O9 P | up | 2.78 | 0.0053 |
| Com_3290_neg | LPE 19:2 | C24 H46 N O7 P | up | 2.45 | 0.0059 |
| Com_5971_neg | L-Glutamic acid monosodium salt | C5 H8 N Na O4 | up | 2.75 | 0.0062 |
| Com_4935_neg | Adenosine 5'-monophosphate | C10 H14 N5 O7 P | down | 0.55 | 0.0073 |
| Com_2410_neg | Nonadecanoic acid | C19 H38 O2 | down | 0.40 | 0.0088 |
| Com_12452_neg | Dihydroroseoside | C19 H32 O8 | up | 5.77 | 0.0097 |
| Com_7201_neg | 12-oxo Leukotriene B4 | C20 H30 O4 | down | 0.49 | 0.0102 |
| Com_2115_neg | Ofloxacin impurity E | C17 H18 F N3 O4 | up | 1.89 | 0.0116 |
| Com_3420_neg | LPS 22:4 | C28 H48 N O9 P | up | 1.96 | 0.0126 |
| Com_149_neg | Taurine | C2 H7 N O3 S | up | 1.76 | 0.0149 |
| Com_7159_neg | LPS 16:1 | C22 H42 N O9 P | up | 3.55 | 0.0152 |
| Com_58_neg | Cholic acid | C24 H40 O5 | down | 0.02 | 0.0155 |
| Com_1090_neg | Taurochenodeoxycholic Acid (sodium salt) | C26 H45 N O6 S | up | 4.57 | 0.0156 |
| Com_2540_neg | Ornithine | C5 H12 N2 O2 | up | 1.78 | 0.0159 |
| Com_1816_neg | Taurolithocholic acid sodium salt | C26 H44 N Na O5 S | up | 3.04 | 0.0165 |
| Com_1249_neg | LPG 16:0 | C22 H45 O9 P | up | 1.50 | 0.0168 |
| Com_2678_neg | 6-Aminonicotinamide | C6 H7 N3 O | up | 1.74 | 0.0171 |
| Com_1110_neg | Taurochenodeoxycholic acid | C26 H45 N O6 S | up | 4.23 | 0.0173 |
| Com_234_neg | Taurocholic acid | C26 H45 N O7 S | up | 3.47 | 0.0185 |
| Com_12671_neg | Protectin D1 | C22 H32 O4 | down | 0.45 | 0.0191 |
| Com_2069_neg | 2-Aminoadipic acid | C6 H11 N O4 | up | 2.31 | 0.0194 |
| Com_4804_neg | L-(+)-Tartaric acid | C4 H6 O6 | up | 2.00 | 0.0195 |
| Com_6203_neg | LPS 17:1 | C23 H44 N O9 P | up | 2.56 | 0.0214 |
| Com_893_neg | Cytidine | C9 H13 N3 O5 | up | 2.22 | 0.0215 |
| Com_10836_neg | Lithocholic Acid | C24 H40 O3 | down | 0.17 | 0.0217 |
| Com_4969_neg | 8-Iso prostaglandin A2 | C20 H30 O4 | down | 0.46 | 0.0225 |
| Com_5531_neg | LPS 16:0 | C22 H44 N O9 P | up | 2.30 | 0.0260 |
| Com_449_neg | Dibenzoyl Thiamine | C26 H26 N4 O4 S | up | 1.59 | 0.0315 |
| Com_1028_neg | D-Glucosamine | C6 H13 N O5 | up | 1.52 | 0.0316 |
| Com_13424_neg | 5-oxo-1-[4-(trifluoromethyl)benzyl]pyrrolidine-3-carboxylic acid | C13 H12 F3 N O3 | up | 10.22 | 0.0333 |
| Com_2048_neg | SM (d14:1/20:0) | C39 H79 N2 O6 P | up | 3.01 | 0.0348 |
| Com_2168_neg | 2-Hydroxy-2-methylbutanoic acid | C5 H10 O3 | up | 1.99 | 0.0378 |
| Com_515_neg | Adenosine diphosphate ribose | C15 H23 N5 O14 P2 | up | 3.29 | 0.0384 |
| Com_152_neg | LPS 18:1 | C24 H46 N O9 P | up | 2.48 | 0.0407 |
| Com_879_neg | Mevalonic acid | C6 H12 O4 | down | 0.34 | 0.0409 |
| Com_10981_neg | N-Acetyl-D-tryptophan | C13 H14 N2 O3 | up | 2.95 | 0.0413 |
| Com_3260_neg | Arachidic acid | C20 H40 O2 | down | 0.16 | 0.0415 |
| Com_13095_neg | 3'-Dephosphocoenzyme A | C21 H35 N7 O13 P2 S | up | 2.40 | 0.0426 |
| Com_2458_neg | Uridine 5'-diphosphogalactose | C15 H24 N2 O17 P2 | down | 0.30 | 0.0433 |
| Com_383_neg | LPC 16:1 | C24 H48 N O7 P | up | 1.95 | 0.0449 |

Note that “∞”means this protein is only expressed in HCD mutton, while “–”means this protein is only expressed in control mutton.

**Table S4.** Differentially abundant compounds of mutton longissimus dorsi between control and high-concentrate diet groups in positive ion mode.

| Compound ID | Compound name | Formula | Trend | Fold change | P-value |
| --- | --- | --- | --- | --- | --- |
| Com_9121_pos | Ursodeoxycholic acid | C24 H40 O4 | down | 0.33 | 0.0000 |
| Com_1262_pos | Ecgonine | C9 H15 N O3 | up | 4.45 | 0.0000 |
| Com_1462_pos | Hippuric acid | C9 H9 N O3 | up | 11.27 | 0.0000 |
| Com_15281_pos | Muramic acid | C9 H17 N O7 | down | 0.08 | 0.0001 |
| Com_2424_pos | Ecgonine methyl ester | C10 H17 N O3 | up | 6.93 | 0.0001 |
| Com_19059_pos | ethyl 2-amino-8H-indeno[2,1-b]thiophene-3-carboxylate | C14 H13 N O2 S | down | 0.08 | 0.0001 |
| Com_1218_pos | 4,4'-dimethoxy[1,1'-biphenyl]-2-carbonitrile | C15 H13 N O2 | up | 2.76 | 0.0001 |
| Com_14621_pos | 3-(1-benzylpiperidin-4-yl)-3H-[1,2,3]triazolo[4,5-b]pyridine | C17 H19 N5 | up | 5.66 | 0.0002 |
| Com_3875_pos | 1-(3,4-dimethoxyphenyl)ethan-1-one oxime | C10 H13 N O3 | down | 0.39 | 0.0003 |
| Com_1418_pos | PB-22 N-(4-Hydroxypentyl)-3-carboxyindole metabolite | C14 H17 N O3 | up | 3.96 | 0.0004 |
| Com_20634_pos | 2-(tert-butyl)-6,7-dimethoxy-4H-3,1-benzoxazin-4-one | C14 H17 N O4 | up | 3.68 | 0.0004 |
| Com_12751_pos | 3-(3,5-dioxo-2,3,4,5-tetrahydro-1,2,4-triazin-6-yl)propanoic acid | C6 H7 N3 O4 | up | 15.25 | 0.0004 |
| Com_16580_pos | 3,4-Methylenedioxy-α-pyrrolidinopropiophenone | C14 H17 N O3 | up | 3.58 | 0.0006 |
| Com_2252_pos | PC (22:6e/15:0) | C45 H80 N O7 P | up | 3.33 | 0.0008 |
| Com_7890_pos | 8-Hydroxyquinoline | C9 H7 N O | up | 4.58 | 0.0008 |
| Com_282_pos | N1-[4-hydroxy-6-(methoxymethyl)pyrimidin-2-yl]acetamide | C8 H11 N3 O3 | up | 1.79 | 0.0013 |
| Com_23976_pos | 12-oxo Phytodienoic Acid | C18 H28 O3 | down | 0.40 | 0.0013 |
| Com_14233_pos | 2-oxa-4-azatetracyclo[6.3.1.1~6,10~.0~1,5~]tridecan-3-one | C11 H15 N O2 | up | 1.78 | 0.0014 |
| Com_1227_pos | Phenylacetylglycine | C10 H11 N O3 | up | 3.69 | 0.0018 |
| Com_31_pos | Betaine | C5 H11 N O2 | up | 1.57 | 0.0019 |
| Com_1436_pos | Linolelaidic Acid (C18:2N6T) | C18 H32 O2 | up | 2.45 | 0.0019 |
| Com_12732_pos | (-)-Epigallocatechin | C15 H14 O7 | up | 2.14 | 0.0024 |
| Com_13323_pos | Pilocarpine | C11 H16 N2 O2 | up | 5.39 | 0.0036 |
| Com_2152_pos | 1-[2-(1H-imidazol-5-yl)ethyl]pyrrolidine-2,5-dione | C9 H11 N3 O2 | up | 1.57 | 0.0047 |
| Com_886_pos | PLK | C17 H32 N4 O4 | up | 2.41 | 0.0058 |
| Com_10264_pos | Lysope 18:1 | C23 H46 N O7 P | up | 1.76 | 0.0063 |
| Com_22176_pos | 3'-Adenosine monophosphate (3'-AMP) | C10 H14 N5 O7 P | up | 2.09 | 0.0064 |
| Com_801_pos | (5ξ,9ξ,16ξ)-17-Hydroxykauran-19-oic acid | C20 H32 O3 | up | 2.60 | 0.0088 |
| Com_23_pos | ACar 18:2 | C25 H46 N O4 | down | 0.33 | 0.0093 |
| Com_471_pos | Epigallocatechin | C15 H14 O7 | up | 2.11 | 0.0109 |
| Com_39_pos | ACar 18:0 | C25 H50 N O4 | down | 0.30 | 0.0110 |
| Com_9251_pos | Androsterone | C19 H30 O2 | down | 0.26 | 0.0110 |
| Com_3962_pos | D-Ala-D-Ala | C6 H12 N2 O3 | up | 1.81 | 0.0123 |
| Com_5165_pos | Dehydroepiandrosterone (DHEA) | C19 H28 O2 | down | 0.27 | 0.0129 |
| Com_235_pos | 7-Ketolithocholic acid | C24 H38 O4 | down | 0.02 | 0.0134 |
| Com_442_pos | L-Glutathione oxidized | C20 H32 N6 O12 S2 | up | 2.11 | 0.0140 |
| Com_3086_pos | PE (6:0/13:1) | C24 H46 N O8 P | up | 2.15 | 0.0145 |
| Com_814_pos | Ala-Gln | C8 H15 N3 O4 | down | 0.63 | 0.0153 |
| Com_2035_pos | LPE 22:4 | C27 H48 N O7 P | up | 1.99 | 0.0156 |
| Com_12213_pos | PE (3:0/13:1) | C21 H40 N O8 P | up | 1.88 | 0.0160 |
| Com_2646_pos | PC (20:5e/15:1) | C43 H76 N O7 P | up | 4.12 | 0.0163 |
| Com_10542_pos | Gramine | C11 H14 N2 | up | 1.90 | 0.0164 |
| Com_97_pos | ACar 17:0 | C24 H48 N O4 | down | 0.31 | 0.0173 |
| Com_13310_pos | methyl 6-{[4-(trifluoromethyl)anilino]carbonyl}nicotinate | C15 H11 F3 N2 O3 | up | 2.69 | 0.0176 |
| Com_374_pos | ACar 20:1 | C27 H52 N O4 | down | 0.15 | 0.0180 |
| Com_4815_pos | PC (4:0/13:1) | C25 H48 N O8 P | up | 6.20 | 0.0183 |
| Com_1170_pos | ACar 19:0 | C26 H52 N O4 | down | 0.15 | 0.0183 |
| Com_333_pos | ACar 18:3 | C25 H44 N O4 | down | 0.31 | 0.0193 |
| Com_2900_pos | ACar 11:0 | C18 H36 N O4 | down | 0.58 | 0.0210 |
| Com_6881_pos | Lysopc 20:4 | C28 H50 N O7 P | up | 2.00 | 0.0217 |
| Com_16297_pos | LysoPC 10:0 | C18 H38 N O7 P | down | 0.41 | 0.0226 |
| Com_10_pos | Palmitoylcarnitine | C23 H45 N O4 | down | 0.58 | 0.0232 |
| Com_18144_pos | ADP-ribose | C15 H23 N5 O14 P2 | up | 3.78 | 0.0234 |
| Com_24472_pos | MMH | C16 H27 N5 O4 S2 | down | 0.56 | 0.0236 |
| Com_1022_pos | ACar 20:0 | C27 H54 N O4 | down | 0.12 | 0.0246 |
| Com_21506_pos | RPK | C17 H33 N7 O4 | down | 0.22 | 0.0248 |
| Com_3643_pos | ACar 22:1 | C29 H56 N O4 | down | 0.15 | 0.0258 |
| Com_8159_pos | PC (20:3e/2:0) | C30 H56 N O7 P | up | 4.21 | 0.0259 |
| Com_187_pos | LPE 18:1 | C23 H46 N O7 P | up | 1.73 | 0.0264 |
| Com_12680_pos | P-Aminohippuric Acid | C9 H10 N2 O3 | down | 0.38 | 0.0277 |
| Com_11369_pos | TKK | C16 H33 N5 O5 | up | 2.47 | 0.0278 |
| Com_11796_pos | PC (18:4e/3:0) | C29 H52 N O7 P | up | 3.22 | 0.0280 |
| Com_17094_pos | Cortisone | C21 H28 O5 | down | 0.51 | 0.0287 |
| Com_16500_pos | RNK | C16 H32 N8 O5 | up | 2.07 | 0.0304 |
| Com_198_pos | ACar 17:1 | C24 H46 N O4 | down | 0.51 | 0.0313 |
| Com_1100_pos | LPE 16:1 | C21 H42 N O7 P | up | 3.52 | 0.0326 |
| Com_4711_pos | PC (20:2/22:6) | C50 H84 N O8 P | down | 0.49 | 0.0336 |
| Com_7796_pos | Proscillaridin A | C30 H42 O8 | down | 0.32 | 0.0349 |
| Com_10417_pos | 4-[3-(benzylamino)butyl]-2-methoxyphenol | C18 H23 N O2 | down | 0.51 | 0.0369 |
| Com_16560_pos | PC (16:1e/9:0) | C33 H66 N O7 P | down | 0.19 | 0.0388 |
| Com_5776_pos | 2-morpholino-1-phenyl-1-ethanol | C12 H17 N O2 | down | 0.45 | 0.0398 |
| Com_18342_pos | PC (20:5e/21:2) | C49 H86 N O7 P | down | 0.23 | 0.0407 |
| Com_1026_pos | Ouabain | C29 H44 O12 | down | 0.01 | 0.0422 |
| Com_1047_pos | L-(+)-Citrulline | C6 H13 N3 O3 | up | 1.96 | 0.0428 |
| Com_1296_pos | 2-Linoleoyl glycerol | C21 H38 O4 | up | 2.22 | 0.0433 |
| Com_13974_pos | 5beta-Androstane-3,17-dione | C19 H28 O2 | down | 0.40 | 0.0442 |
| Com_7332_pos | ACar 22:2 | C29 H54 N O4 | down | 0.15 | 0.0460 |
| Com_11697_pos | Ribulose-5-phosphate | C5 H11 O8 P | up | 4.32 | 0.0462 |
| Com_20377_pos | DL-Dipalmitoylphosphatidylcholine | C40 H80 N O8 P | up | 2.08 | 0.0469 |
| Com_7131_pos | PC (18:3/20:5) | C46 H76 N O8 P | down | 0.59 | 0.0472 |
| Com_21672_pos | Linalool | C10 H18 O | down | 0.47 | 0.0472 |
| Com_1248_pos | N-Methyllysine | C7 H16 N2 O2 | up | 1.95 | 0.0478 |
| Com_11656_pos | 13,14-dihydro-15-keto Prostaglandin A2 | C20 H30 O4 | down | 0.44 | 0.0479 |

**Table S5.** The KEGG functional enrichments of differential metabolites in negative ion mode.

| Map ID | Describe | No. of protein | P-value | Pivotal compounds |
| --- | --- | --- | --- | --- |
| map00480 | Primary bile acid biosynthesis | 4 | 0.0011 | Taurochenodeoxycholic acid, Taurine, Taurocholic acid, Cholic acid |
| map00830 | Taurine and hypotaurine metabolism | 3 | 0.0180 | Taurine, Taurocholic acid, L-Glutamic acid |
| map04966 | Cholesterol metabolism | 2 | 0.0493 | Taurochenodeoxycholic acid, Taurocholic acid |
| map00982 | Bile secretion | 4 | 0.0588 | Lithocholic Acid, Taurochenodeoxycholic acid, Taurocholic acid, Cholic acid |
| map01524 | D-Glutamine and D-glutamate metabolism | 1 | 0.1383 | L-Glutamic acid |
| map00910 | Nitrogen metabolism | 1 | 0.1383 | L-Glutamic acid |
| map05204 | cAMP signaling pathway | 1 | 0.1383 | Adenosine 5'-monophosphate |
| map00980 | FoxO signaling pathway | 1 | 0.1383 | Adenosine 5'-monophosphate |
| map04964 | mTOR signaling pathway | 1 | 0.1383 | Adenosine 5'-monophosphate |
| map04976 | PI3K-Akt signaling pathway | 1 | 0.1383 | Adenosine 5'-monophosphate |

**Table S6.** The KEGG functional enrichments of differential metabolites in positive ion mode.

| Map ID | Describe | No. of protein | P-value | Pivotal compounds |
| --- | --- | --- | --- | --- |
| map00360 | Phenylalanine metabolism | 2 | 0.0861 | Phenylacetylglycine, Hippuric acid |
| map00140 | Steroid hormone biosynthesis | 3 | 0.1670 | 5beta-Androstane-3,17-dione, Cortisone, Androsterone |
| map00260 | Glycine, serine and threonine metabolism | 1 | 0.2892 | Betaine |
| map04960 | Aldosterone-regulated sodium reabsorption | 1 | 0.2892 | Cortisone |
| map02010 | ABC transporters | 1 | 0.5008 | Betaine |
| map05200 | Pathways in cancer | 1 | 0.5008 | Cortisone |
| map05215 | Prostate cancer | 1 | 0.5008 | Cortisone |
| map04976 | Bile secretion | 1 | 1.0000 | Ouabain |
| map00230 | Purine metabolism | 1 | 1.0000 | ADP-ribose |


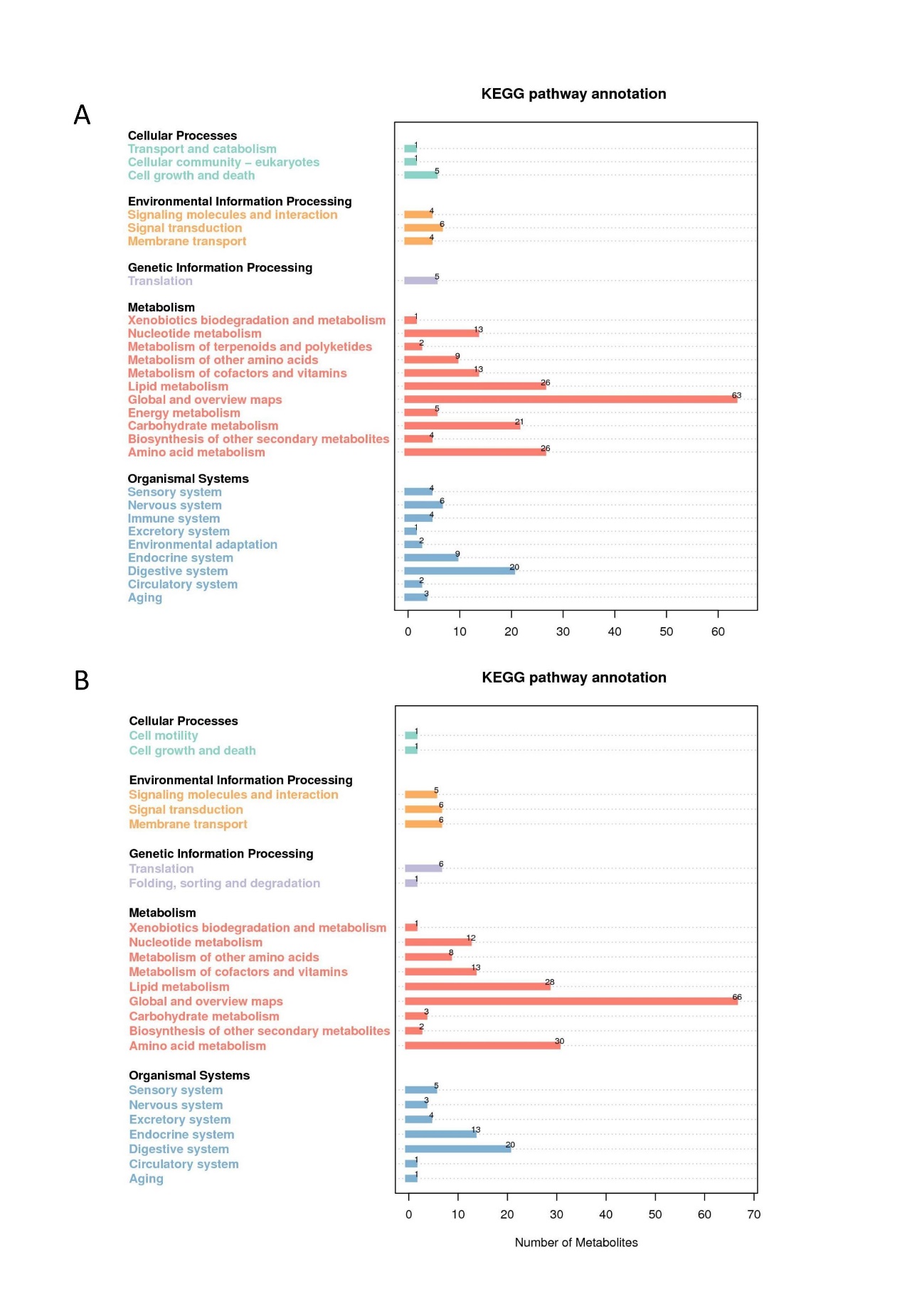


**Figure S1.** KEGG annotation classification of differentially abundant metabolites in HCD mutton in the ESI- (A) and ESI+ modes (B).


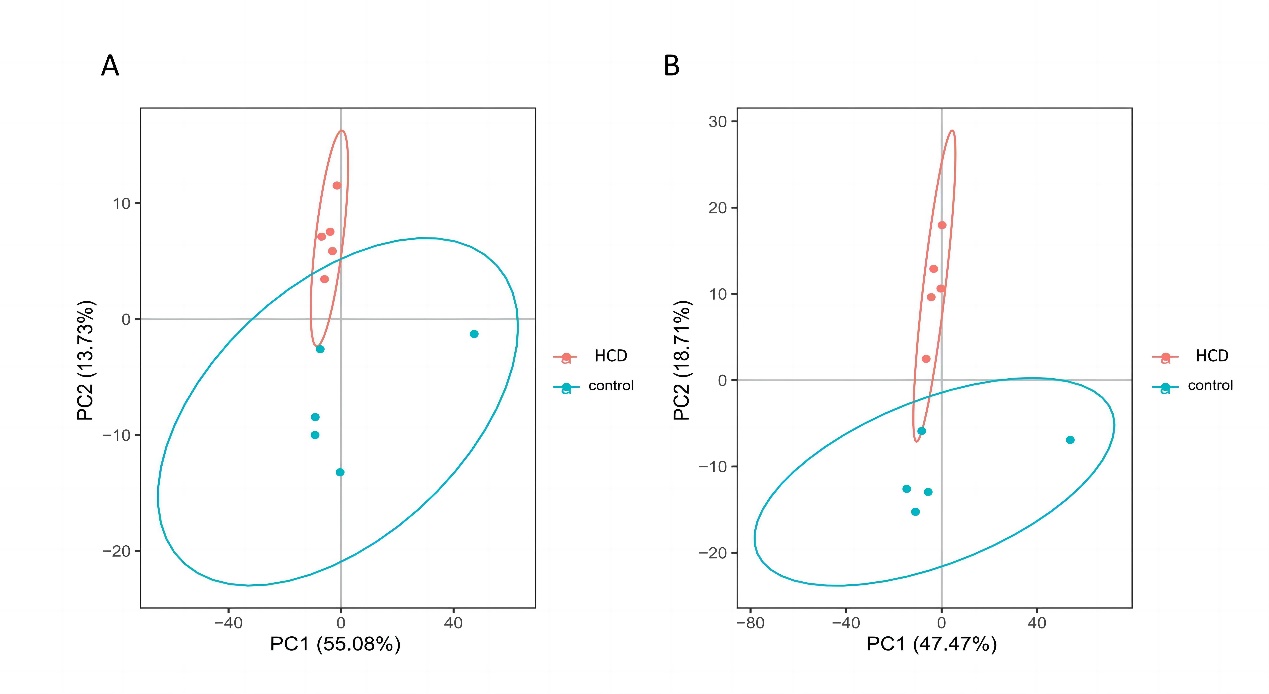


**Figure S2.** PCA scores plot of control and HCD mutton in the ESI- (A) and ESI+ modes (B).


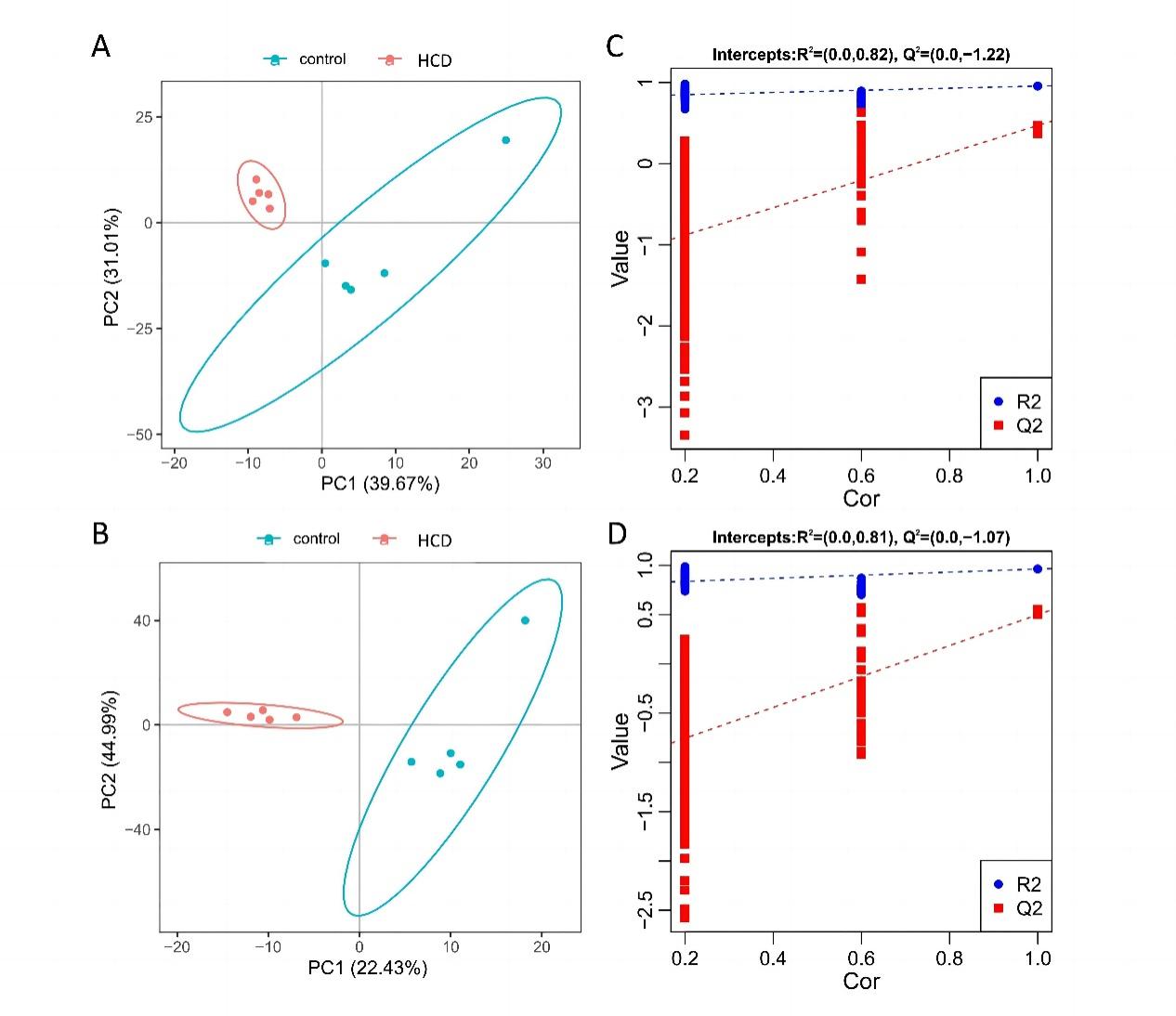


**Figure S3.** PLS-DA score plot along with response ranking test of metabolites of HCD mutton in the ESI- (A) and ESI+ modes (B)
